# Clinical manifestations and immunomodulatory treatment experiences in psychiatric patients with suspected autoimmune encephalitis: a case series of 91 patients from Germany

**DOI:** 10.1038/s41380-021-01396-4

**Published:** 2022-01-19

**Authors:** Dominique Endres, Eva Lüngen, Alkomiet Hasan, Michael Kluge, Sabrina Fröhlich, Jan Lewerenz, Tom Bschor, Ida Sibylle Haußleiter, Georg Juckel, Florian Then Bergh, Barbara Ettrich, Lisa Kertzscher, Tatiana Oviedo-Salcedo, Robert Handreka, Martin Lauer, Klaas Winter, Norbert Zumdick, Anna Drews, Jost Obrocki, Yavor Yalachkov, Anna Bubl, Felix von Podewils, Udo Schneider, Kristina Szabo, Margarete Mattern, Alexandra Philipsen, Katharina Domschke, Klaus-Peter Wandinger, Alexandra Neyazi, Oliver Stich, Harald Prüss, Frank Leypoldt, Ludger Tebartz van Elst

**Affiliations:** 1 Section for Experimental Neuropsychiatry, Department of Psychiatry and Psychotherapy, Medical Center - University of Freiburg, Faculty of Medicine, University of Freiburg, Freiburg, Germany; 2 Department of Psychiatry and Psychotherapy, Medical Center - University of Freiburg, Faculty of Medicine, University of Freiburg, Freiburg, Germany; 3 Department of Psychiatry, Psychotherapy and Psychosomatics, Medical Faculty, University of Augsburg, BKH Augsburg, Augsburg, Germany; 4 Department of Psychiatry and Psychotherapy, University Hospital, Munich, Germany; 5 Department of Psychiatry and Psychotherapy, University of Leipzig, Leipzig, Germany; 6 Department of Psychiatry and Psychotherapy, Ludwig-Noll-Krankenhaus, Kassel, Germany; 7 Department of Neurology and Clinical Neurophysiology, DRK Hospital Nordhessen, Kassel, Germany; 8 Department of Neurology, University of Ulm, Ulm, Germany; 9 Department of Psychiatry and Psychotherapy, University Hospital Dresden, Dresden, Germany; 10 Department of Psychiatry, LWL-University Hospital, Ruhr University Bochum, Bochum, Germany; 11 Department of Neurology, University of Leipzig, Leipzig, Germany; 12 Department of Neurology, Carl-Thiem-Klinikum Cottbus, Cottbus, Germany; 13 Department of Psychiatry, Psychosomatics and Psychotherapy, Center of Mental Health, University Hospital Würzburg, Würzburg, Germany; 14 Department of Psychiatry and Psychotherapy, Carl-Friedrich-Flemming-Klinik, Helios Kliniken Schwerin, Schwerin, Germany; 15 Department of Psychiatry and Psychotherapy Medicine, St. Marien-Hospital Hamm, Hamm, Germany; 16 Department of Psychiatry and Psychotherapy, Vinzenz von Paul Hospital Rottenmünster, Rottweil, Germany; 17 Department of Psychiatry, Psychotherapy and Psychosomatic Medicine, Regio Klinikum Elmshorn, Elmshorn, Germany; 18 Department of Neurology, University Hospital/Goethe University, Frankfurt/Main, Germany; 19 Department of Psychiatry and Psychotherapy, University of Saarland, Homburg/Saar, Germany; 20 Department of Neurology, University Medicine Greifswald, Greifswald, Germany; 21 Department of Psychiatry and Psychotherapy, Ruhr-University Bochum Campus-OWL Lübbecke, Lübbecke, Germany; 22 Department of Neurology and Mannheim Center for Translational Neuroscience, Medical Faculty Mannheim, Heidelberg University, Mannheim, Germany; 23 Department of General Psychiatry, Center for Psychosocial Medicine, University Hospital Heidelberg, Heidelberg, Germany; 24 Department of Psychiatry and Psychotherapy, University of Bonn, Bonn, Germany; 25 Center for Basics in NeuroModulation, Faculty of Medicine, University of Freiburg, Freiburg, Germany; 26 Neuroimmunology Section, Institute of Clinical Chemistry, University Hospital Schleswig-Holstein Kiel/Lübeck, Lübeck, Germany; 27 Department of Psychiatry, Social Psychiatry and Psychotherapy, Hannover Medical School, Hannover, Germany; 28 Neurology, Medical Care Center, Konstanz, Germany; 29 Department of Neurology, Medical Center, Faculty of Medicine, University of Freiburg, Freiburg, Germany; 30 Department of Neurology and Experimental Neurology, Charité - Universitätsmedizin Berlin, Berlin, Germany; 31 German Center for Neurodegenerative Diseases (DZNE), Berlin, Germany; 32 Department of Neurology, Christian-Albrechts-University Kiel, Kiel, Germany

**Keywords:** Molecular biology, Diagnostic markers

## Abstract

Autoimmune encephalitis (AE) can rarely manifest as a predominantly psychiatric syndrome without overt neurological symptoms. This study’s aim was to characterize psychiatric patients with AE; therefore, anonymized data on patients with suspected AE with predominantly or isolated psychiatric syndromes were retrospectively collected. Patients with readily detectable neurological symptoms suggestive of AE (e.g., epileptic seizures) were excluded. Patients were classified as “probable psychiatric AE (pAE),” if well-characterized neuronal IgG autoantibodies were detected or “possible pAE” (e.g., with detection of nonclassical neuronal autoantibodies or compatible cerebrospinal fluid (CSF) changes). Of the 91 patients included, 21 (23%) fulfilled our criteria for probable (autoantibody-defined) pAE and 70 (77%) those for possible pAE. Among patients with probable pAE, 90% had anti-NMDA receptor (NMDA-R) autoantibodies. Overall, most patients suffered from paranoid-hallucinatory syndromes (53%). Patients with probable pAE suffered more often from disorientation (*p* < 0.001) and impaired memory (*p* = 0.001) than patients with possible pAE. Immunotherapies were performed in 69% of all cases, mostly with high-dose corticosteroids. Altogether, 93% of the patients with probable pAE and 80% of patients with possible pAE reportedly benefited from immunotherapies (*p* = 0.251). In summary, this explorative, cross-sectional evaluation confirms that autoantibody-associated AE syndromes can predominantly manifest as psychiatric syndromes, especially in anti-NMDA-R encephalitis. However, in three out of four patients, diagnosis of possible pAE was based on nonspecific findings (e.g., slight CSF pleocytosis), and well-characterized neuronal autoantibodies were absent. As such, the spectrum of psychiatric syndromes potentially responding to immunotherapies seems not to be limited to currently known autoantibody-associated AE. Further trials are needed.

## Introduction

Autoimmune encephalitis (AE) comprises an emerging group of autoinflammatory diseases of the brain. In many but not all cases, autoantibodies (Abs) targeting neuronal antigens have been discovered in recent years. AEs usually manifest with complex neuropsychiatric syndromes with neurological signs and symptoms, such as epileptic seizures, movement disorders, or focal neurological deficits, and a plethora of psychiatric symptoms [1]. Their discovery, as well as the fact that many of these patients initially have behavioral and psychiatric symptoms, has sparked concerns that underlying autoimmune and inflammatory processes could be more frequent in patients with primarily psychiatric syndromes than previously appreciated. Several case series have analyzed the frequency of isolated psychiatric manifestations in Ab-defined AE and generally found it to be rather infrequent. For example, in a case series on anti-NMDA receptor (NMDA-R) encephalitis, only 4% of the patients (mostly at the time of relapse) did not have associated neurological manifestations [2]. However, numerous single cases with predominant or isolated psychiatric manifestations of AE have been observed, presenting, for example, with the clinical picture of an idiopathic schizophrenia-spectrum disorder [3–7]. The term “autoimmune psychosis” (AP) has recently been proposed for such patients [7]. Cerebrospinal fluid (CSF) analysis plays a prominent role in the diagnostic workup of patients with suspected AE/AP [1, 7]. The detection of well-characterized neuronal Abs in CSF is considered a criterion for definite AP [7]; in addition, inflammatory CSF changes are among the most sensitive markers of AE/AP [1, 3, 7]. CSF routine analysis allows the determination of the white blood cell (WBC) count, as a marker for acute inflammatory processes and whether intrathecal immunoglobulin (Ig) synthesis found in chronic inflammatory diseases of the brain is present, either qualitatively as CSF-specific oligoclonal bands (OCBs, type 2 + 3) or in combination therewith also quantitatively for IgG, IgA, and/or IgM synthesis [8, 9]. In contrast, CSF protein concentration and CSF/blood albumin quotient are markers of blood–CSF barrier function probably reflecting altered CSF flow dynamics and may also be changed in noninflammatory brain disorders [4, 8, 9]. The IgG index can be used to assess quantitative intrathecal IgG synthesis (but it can yield false positive results if the albumin quotient is high) as do the Reiber’s hyperbolic graphs for all the Ig classes [9].

Previous literature studies investigating psychiatric AE (pAE) have reviewed subgroups of published psychiatric cases [3, 10]; however, larger cumulative case collections of unselected patients with psychiatric manifestations and a suspected diagnosis of AE are still lacking. The aim of this study, therefore, was to describe a retrospective cohort of patients considered by treating physicians in a German inpatient setting to have autoimmune psychiatric syndromes.

## Materials and methods

### Patients and data acquisition

Participating institutions were asked to screen patients who were treated as inpatients and fulfilled the inclusion and exclusion criteria. Patient data was then entered in anonymized fashion using an electronic reporting form adapted from GErman NEtwork for Research on AuToimmune Encephalitis (GENERATE; https://generate-net.de/) for specific aspects of patients with psychiatric syndromes (“GENERATE-psych” subdatabase). The data collection was fully anonymized and was approved by the ethics committee of the Faculty of Medicine, University of Freiburg (ethical vote no. 498/15).

### Inclusion and exclusion criteria

For this analysis, only patients with isolated or predominantly psychiatric syndromes (e.g., schizophrenia-spectrum disorders, depression, or pseudodementia/dementia) who showed the following findings suggestive for AE were included if the following criteria were met:

1. Detectable neuronal IgG Abs against cell surface antigens (e.g., NMDA-R/AMPA-R/GABA-B-R/VGKC-complex[C]/LGI1/CASPR2/mGluR1/R5/glycine-R/DPPX/uncharacterized neuropil-specific immunostaining on rat brain) or glial antigens (AQP4/MOG) in serum and/or CSF;
  OR
2. detectable IgG Abs against intracellular neuronal/glial antigens (GAD65/amphiphysin/Yo/Hu/Ri/Ma1/2/Zic4/SOX1/AK5/Ca/ARHGAP2, etc.) in serum and/or CSF;
  OR
3. detectable IgG Abs against thyroid antigens (TPO/TG/TSH receptor) in serum and/or CSF and a clinical response to any immunomodulatory therapy;
  OR
4. inflammatory changes in CSF (WBC count ≥ 5 cells/µl, the detection of isolated OCBs in CSF, increased IgG index, positive MRZ-reaction), or clinically relevant (judged by treating physician) response to any immunomodulatory treatment.

Patients with a clear clinical picture of AE (e.g., with tonic-clonic or focal seizures, noniatrogenic loss of consciousness) were excluded. Movement disorders (e.g., catatonia) did not lead to exclusion if considered related to psychosis or medication by treating physicians. Dementia and pseudodementia syndromes were considered as psychiatric syndromes for this study.

### Subgroup definition

All patients with suspected AE were classified by the authors as “probable pAE” or “possible pAE” based on the provided data. For subgroup analyses, four groups were created based on Ab findings (Table 1).

**Table 1:** Subgroup definitions.

Probable psychiatric autoimmune encephalitis (“probable pAE”)	Possible psychiatric autoimmune encephalitis (“possible pAE”)
Patients with psychiatric syndromes and detection of well-characterized neuronal IgG Abs against cell surface or glial antigens (NMDA-R, LGI1, MOG) in serum and/or CSF.	Patients with psychiatric syndromes and the detection of well-characterized onconeuronal Abs by immunoblot (Yo, Zic4), non-well-characterized neuronal Abs (glycine-R, VGKC-C without LGI1/CASPR2 antibodies, GAD65, uncharacterized neuropil-specific immunostaining on rat/mouse brain immunohistochemistry) or clinical response to immunomodulatory therapies with or without detection of antithyroid Abs or detection of inflammatory CSF pathologies.
Subgroups (based on antibody findings)	
• Patients with pAE associated with antibodies against cell surface antigens. • Patients with pAE associated with antibodies against intracellular antigens. • Patients with probable Hashimoto encephalopathy based on detection of any thyroid antibodies and response to immunotherapy. • Seronegative patients with “potential psychiatric autoimmune syndromes” based on inflammatory CSF alterations and/or a response to a trial of immunotherapy as rated by the treating physician.	

### Statistics

The anonymized data were analyzed using SPSS25 (IBM, New York, USA). Most results were presented descriptively. Subgroup comparisons for categorical variables, such as sex ratio, were carried out using Pearson’s *χ*^2^ test. Dimensional variables, such as age, were compared using independent sample *t*-tests (between two groups) and one-way ANOVAs (between more than two groups). Associations between white matter lesions in magnetic resonance imaging (MRI) and age were analyzed using logistic regression. A *p* value of <0.05 was considered significant. Due to the study’s explorative character, we did not perform corrections for multiple testing. Full test statistics are displayed in the tables.

## Results

### Sociodemographic and clinical characteristics

**Fig. 1:**
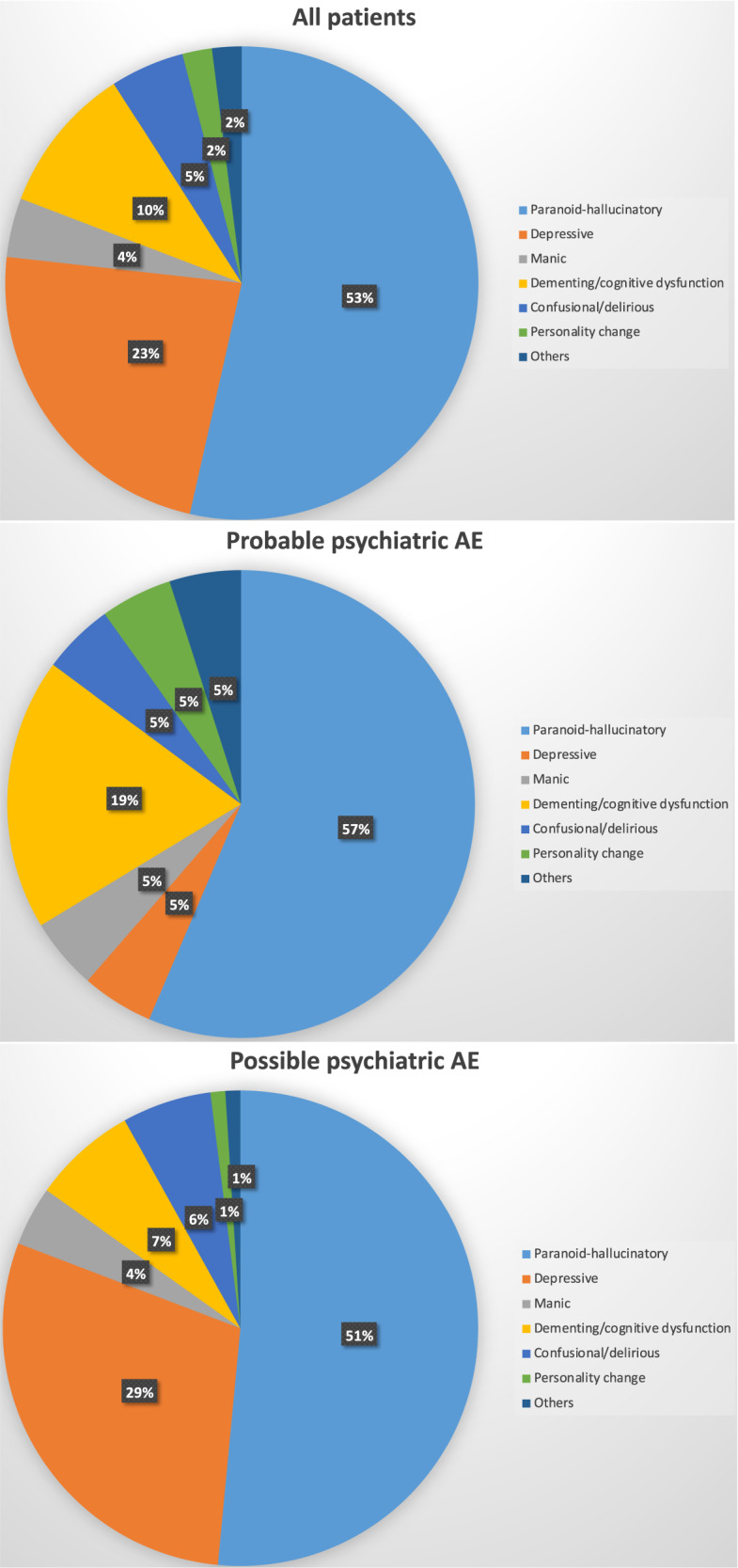
Syndromal findings (presented here is the predominant psychiatric syndrome for each patient). *AE* autoimmune encephalitis. The group of patients with paranoid-hallucinatory syndromes includes all patients with catatonic syndromes.

Altogether, 91 patients were included in the study: 21 (23%) with probable pAE and 70 (77%) with possible pAE (Table 2). In both groups, women were slightly overrepresented (57% in total). Patients with possible pAE tended to be older than patients with probable pAE (*p* = 0.091). Most patients were born in Germany (at least ≈50%). In both groups, the majority of patients suffered from paranoid-hallucinatory syndrome (53% in total), with no significant differences in syndrome distribution between patients with probable and possible pAE (*χ*^2^ = 8.051, *p* = 0.234; Fig. 1). The most common psychiatric symptoms were (1) affective symptoms (in 93% of the patients), (2) altered energy and psychomotor activity (in 87% of the patients), and (3) attention and concentration deficits (in 74% of the patients). A comparison of psychopathological findings revealed significantly more frequent disorientation (*p* < 0.001), impaired memory (*p* = 0.001), and a tendency for more frequent optical hallucinations (*p* = 0.053) among patients with probable pAE than with possible pAE. Autonomic dysregulation was more frequent in patients with probable pAE (*p* = 0.045). With preexisting psychiatric conditions present in 5/19 (26%) of patients with probable AE, these were almost twofold less frequent than in the cohort with possible pAE (34/67; 51%). However, this difference failed to reach statistical significance (*p* = 0.059).

**Table 2:** Demographics and clinical findings.

	All patients (*N* = 91)	Probable psychiatric autoimmune encephalitis (*N* = 21)	Possible psychiatric autoimmune encephalitis (*N* = 70)	Statistics^c^
Demographic information				
Females (%): males (%)	52 (57%): 39 (43%)	14 (67%): 7 (33%)	38 (54%): 32 (46%)	*χ*^2^ = 1.011, *p* = 0.315
Mean age ± SD	40.53 ± 16.05 (*N* = 88)^a^	35.20 ± 17.53 (*N* = 20)	42.10 ± 15.37 (*N* = 68)	*T* = −1.710, *p* = 0.091
Psychometric information				
Disturbance of consciousness	1% (1/90)	0% (0/21)	1% (1/69)	*χ*^2^ = 0.308, *p* = 0.579
Disorientation	21% (17/82)	53% (9/17)	12% (8/65)	*χ*^2^ = 13.539, ***p*** < **0.001**
Attention and concentration deficits	74% (54/73)	71% (10/14)	75% (44/59)	*χ*^2^ = 0.058, *p* = 0.809
Impaired memory	45% (34/75)	81% (13/16)	36% (21/59)	*χ*^2^ = 10.587, ***p*** = **0.001**
Formal thought dysfunction	70% (54/77)	53% (8/15)	74% (46/62)	*χ*^2^ = 2.509, *p* = 0.113
Anxiety and obsessive-compulsive symptoms	44% (31/71)	33% (4/12)	46% (27/59)	*χ*^2^ = 0.626, *p* = 0.429
Delusions	53% (40/76)	60% (9/15)	51% (31/61)	*χ*^2^ = 0.407, *p* = 0.523
Acoustical hallucinations	29% (21/72)	27% (4/15)	30% (17/57)	*χ*^2^ = 0.057, *p* = 0.811
Optical hallucinations	20% (15/74)	38% (6/16)	16% (9/58)	*χ*^2^ = 3.750, *p* = 0.053
Loss of ego-boundaries	23% (15/66)	36% (4/11)	20% (11/55)	*χ*^2^ = 1.398, *p* = 0.237
Affective disorder	93% (77/83)	94% (16/17)	92% (61/66)	*χ*^2^ = 0.058, *p* = 0.810
Altered energy and psychomotor activity	87% (66/76)	93% (14/15)	85% (52/61)	*χ*^2^ = 0.689, *p* = 0.406
Suicidality	26% (19/72)	8% (1/12)	30% (18/60)	*χ*^2^ = 2.417, *p* = 0.120
Behavior harmful to others	6% (4/72)	15% (2/13)	3% (2/59)	*χ*^2^ = 2.921, *p* = 0.087
Sleep disturbance	64% (43/67)	50% (6/12)	67% (37/55)	*χ*^2^ = 1.278, *p* = 0.258
Additional symptoms				
Nonspecific accompanying neurological signs overall	26% (23/89)	24% (5/21)	26% (18/68)	*χ*^2^ = 0.059, *p* = 0.808
Motor symptoms^b^	19% (17/89)	10% (2/21)	22% (15/68)	
Dissociative states	2% (2/89)	10% (2/21)	0% (0/68)	
Hyposmia	1% (1/89)	0% (0/21)	1% (1/68)	
Visual symptoms	2% (2/89)	5% (1/21)	1% (1/68)	
Headache	1% (1/89)	0% (0/21)	1% (1/68)	
Autonomic dysregulation overall	6% (5/86)	15% (3/20)	3% (2/66)	*χ*^2^ = 4.016, ***p*** = **0.045**
Urinary incontinence	1% (1/86)	0% (0/20)	2% (1/66)	
Heavy sweating	1% (1/86)	0% (0/20)	2% (1/66)	
Tachycardia	1% (1/86)	5% (1/20)	0% (0/66)	
Hypertensive crisis	1% (1/86)	5% (1/20)	0% (0/66)	
Syncope	1% (1/86)	5% (1/20)	0% (0/66)	

*Abs* autoantibodies.

^a^Not available from three patients.

^b^Considered to be caused by medication or catatonia by treating physicians.

^c^Statistics refer to the comparison between patients with probable and possible psychiatric autoimmune encephalitis.

Significant changes are marked in bold.

### Diagnostic findings and cancer association

**Fig. 2:**
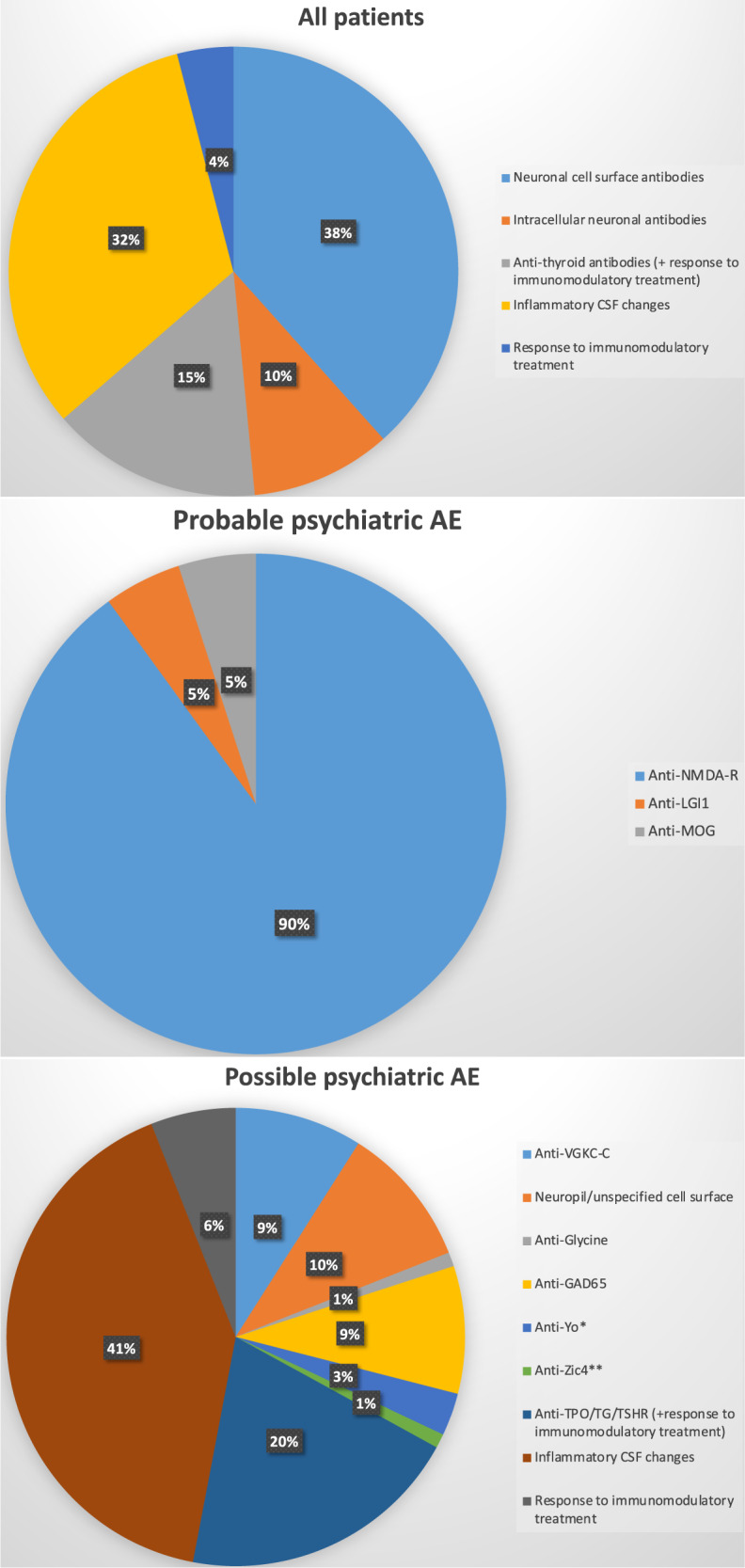
Autoantibody findings. Inflammatory CSF pathologies are coded here only if patients had no additional neuronal antibodies. For a small subgroup of patients only response to immunomodulatory treatment was documented. *Anti-Yo antibodies were tested in one patient using an immunoblot and in indirect immunofluorescence, in the second anti-Yo positive patient, these antibodies were positive in two immunoblots. **Unfortunately, the antibody measurement method in the anti-Zic4 positive patient was not documented. AE autoimmune encephalitis, CSF cerebrospinal fluid.

Neuronal Ab investigation was documented for CSF in 81% and for serum in 89% of all patients. Ab analyses were performed more often in the patient group with probable pAE (95% in CSF and 95% in serum) compared to patients with possible pAE (77% in CSF and 87% in serum). Within all investigated patients, Ab detection was reported in 39% in CSF and in 65% in serum (of the investigated patients). In the probable pAE group (*N* = 21), 19 patients were positive for anti-NMDA-R Abs (90%; 21% of all patients) and one patient each for anti-LGI1 Abs (5%) and anti-MOG Abs (5%; Fig. 2). In 15 of all 21 patients with probable pAE (71%) the Abs were detected in CSF (not analyzed in the CSF of one patient—this corresponds to a positive result in 15 of 20 [75%] of the tested patients). In 14 of 19 the patients positive for anti-NMDA-R Abs (74%; not tested in the CSF of one patient—this corresponds to a positive result in 14 of 18 [78%] of the tested patients), the Abs were positive in CSF. Anti-LGI1 Abs were documented to be positive in the CSF of the one patient, anti-MOG Abs were tested negative in the CSF of the other patient.

Pathological findings in CSF (CSF results reported from 95% of all patients) were common (in 77%), inflammatory CSF pathologies were identified in 53%. The most frequent CSF pathology was pleocytosis, which was found in 42% of all patients. MRI changes (imaging results reported in 97%) were detected in 57%. Presence of white matter lesions on MRI was not associated with age (*F* = 2.034, *p* = 0.158). [18F]fluorodeoxyglucose positron emission tomography (FDG-PET) pathologies were described in 56% (only results available in 30%) and electroencephalography (EEG) alterations in 40% of all investigated patients (results available from 82%). No significant differences between the patients with probable and possible pAE were found in overall CSF, MRI, EEG, and FDG-PET alterations. Malignancies were detected in 3% of all patients (Table 3).

**Table 3:** Diagnostic findings and cancer association.

	All patients (*N* = 91)	Probable psychiatric autoimmune encephalitis (*N* = 21)	Possible psychiatric autoimmune encephalitis (*N* = 70)	Statistics^d^
Laboratory findings				
CSF results reported	95% (86/91)	95% (20/21)	94% (66/70)	–
CSF overall altered^a^	77% (66/86)	85% (17/20)	74% (49/66)	*χ*^2^ = 0.995, *p* = 0.318
Inflammatory CSF changes (increased WBC count and/or OCBs in CSF)	53% (46/86)	65% (13/20)	50% (33/66)	*χ*^2^ = 1.388, *p* = 0.239
WBC count ↑	42% (36/86)	60% (12/20)	36% (24/66)	
Protein levels ↑	41% (35/86)	35% (7/20)	42% (28/66)	
Albumin quotient ↑	35% (23/66)	36% (5/14)	35% (18/52)	
IgG index ↑	20% (17/86)	0% (0/20)	26% (17/66)	
OCBs in CSF/OCBs in CSF and serum	23% (18/77)/13% (10/77)	21% (3/14)/36% (5/14)	24% (15/63)/8% (5/63)	
Instrument based diagnostics				
MRI results reported	97% (88/91)	95% (20/21)	97% (68/70)	–
MRI overall alterations	57% (50/88)	45% (9/20)	60% (41/68)	*χ*^2^ = 1.473, *p* = 0.225
White matter changes	49% (43/88)	45% (9/20)	50% (34/68)	
Generalized cortical atrophy	2% (2/88)	0% (0/20)	3% (2/68)	
Localized cortical atrophy	7% (6/88)	10% (2/20)	6% (4/68)	
Post-ischemic defects	1% (1/88)	0% (0/20)	1% (1/68)	
Anatomical variants (e.g., cysts)	5% (4/88)	0% (0/20)	6% (4/68)	
Others	7% (6/88)	5% (1/20)	7% (5/68)	
EEG results reported	82% (75/91)	76% (16/21)	84% (59/70)	–
EEG overall alterations^b^	40% (30/75)	38% (6/16)	41% (24/59)	*χ*^2^ = 0.053, *p* = 0.818
Epileptic activity	4% (3/75)	0% (0/16)	5% (3/59)	
Slowing	39% (29/75)	38% (6/16)	39% (23/59)	
FDG-PET results reported	30% (27/91)	14% (3/21)	34% (24/70)	–
FDG-PET overall alterations	56% (15/27)	67% (2/3)	54% (13/24)	*χ*^2^ = 0.169, *p* = 0.681
Cancer association				
Malignancies^c^	3% (3/91)	10% (2/21)	1% (1/70)	*χ*^2^ = 3.321, *p* = 0.068

Some patients were documented as having received an examination; however, the corresponding findings were not entered into the database. These findings were assessed here as “not reported findings” and therefore do not appear in the table. If several findings were abnormal (e.g., the MRI showed two alterations), then both pathologies were coded.

*MRI* magnetic resonance imaging, *Abs* autoantibodies, *BBB* blood–brain barrier, *EEG* electroencephalography, *FDG-PET* [18F]Fluorodeoxyglucose positron emission tomography, *CSF* cerebrospinal fluid, *WBC* white blood cell, *IgG* immunoglobulin G.

^a^Defined as increased WBC count, increased protein concentration/albumin quotient, increased lactate, increased IgG index, oligoclonal bands (including identical bands in serum and CSF).

^b^Thirty patients had epileptic activity and slowing, two patients also showed anterior pronounced basic activity.

^c^Type of Malignancy: Anal carcinoma (*N* = 1), prostate carcinoma (*N* = 1), cold thyroid node (*N* = 1).

^d^Statistics refer to the comparison between patients with probable and possible psychiatric autoimmune encephalitis.

### Immunomodulatory treatment approaches and responses

Altogether, 69% of the patients received immunotherapies, with no significant group differences between patients with probable and possible pAE (*p* = 0.804). A total of 93% of the patients with probable pAE and 80% of the patients with possible pAE were considered to benefit from immunomodulatory treatment by treating physicians (outcome not documented for five patients), without a significant group difference (*p* = 0.251). Steroids were used most frequently (in 57% at high dose, in 38% at low dose), followed by plasmapheresis/immunoadsorption (in 13%) or intravenous immunoglobulins (IVIGs; in 11%). Altogether, 16% of the patients were treated with long-term steroids and/or steroid-sparing agents. Notably, more patients with probable pAE than with possible pAE were treated with rituximab (3/21 [14%] versus 1/70 [1%]) and vice versa for long-term steroids or steroid-sparing agents (1/21 [5%] versus 14/70 [20%]). Most patients also received psychopharmacological drugs (93%) (Table 4).

**Table 4:** Treatment approaches and responses.

	All patients (*N* = 91)	Probable psychiatric autoimmune encephalitis (*N* = 21)	Possible psychiatric autoimmune encephalitis (*N* = 70)	Statistics^c^
Immunomodulatory treatment				
Immunotherapies overall^a^	69% (63/91)	71% (15/21)	69% (48/70)	*χ*^2^ = 0.062, *p* = 0.804
Overall improvement^b^	83% (48/58)	93% (13/14)	80% (35/44)	*χ*^2^ = 1.319, *p* = 0.251
High-dose i.v. steroids	57% (52/91)	67% (14/21)	54% (38/70)	-
Improvement^b^	72% (34/47)	67% (8/12)	74% (26/35)	
“Low dose” oral steroids	38% (35/91)	29% (6/21)	41% (29/70)	-
Improvement^b^	81% (21/26)	75% (3/4)	82% (18/22)	
Plasmapheresis/immune-adsorption	13% (12/91)	14% (3/21)	13% (9/70)	-
Improvement	67% (6/9)	50% (1/2)	71% (5/7)	
Intravenous immunoglobulins	11% (10/91)	43% (9/21)	1% (1/70)	-
Improvement^b^	88% (7/8)	100% (7/7)	0% (0/1)	
Rituximab	4% (4/91)	14% (3/21)	1% (1/70)	-
Improvement	100% (4/4)	100% (3/3)	100% (1/1)	
Long-term immunotherapy	16% (15/91)	5% (1/21)	20% (14/70)	*χ*^2^ = 2.024, *p* = 0.155
Steroids	33% (5/15)	0% (0/1)	36% (5/14)	
Azathioprine	47% (7/15)	100% (1/1)	43% (6/14)	
Methotrexate	13% (2/15)	0% (0/1)	14% (2/14)	
Other	7% (1/15)	0% (0/1)	7% (1/14)	
Psychopharmacological medication				
Psychotropic drugs overall	93% (85/91)	95% (20/21)	93% (65/70)	*χ*^2^ = 0.149, *p* = 0.700
Antipsychotics	86% (71/85)	80% (16/20)	85% (55/65)	
Antidepressants	48% (41/85)	40% (8/20)	51% (33/65)	
Anticonvulsants	42% (36/85)	65% (13/20)	35% (23/65)	

In five patients, outcome after immunotherapy was not documented. Therefore, there is more data on treatments overall than on outcome.

*i.v.* intravenous.

^a^Steroids, plasma exchange/immunoadsorption, intravenous immunoglobulins, rituximab, cyclosphosphamide.

^b^There were patients in whom the nature of the treatment was known but the response remained unclear.

^c^Statistics refer to the comparison between patients with probable and possible psychiatric autoimmune encephalitis.

### Subgroup analyses

Overall, 35 patients (38%) had Abs against neuronal cell surface/glial antigens, and in nine patients (10%) Abs against intracellular neuronal antigens were detected. Fourteen patients (15%) fulfilled criteria for probable Hashimoto encephalopathy, and 33 seronegative patients (36%) suffered from “potential psychiatric autoimmune syndromes” based on suspicious findings in MRI, CSF, or EEG analyses or a response to immunotherapy trials (Supplemental Table 1). Significant differences were found in sex ratios (*p* = 0.021) with 93% of the patients with probable Hashimoto encephalopathy being female. No significant differences in age (*p* = 0.365), clinical syndromes (*p* = 0.322), imaging/EEG findings (MRI: *p* = 0.376; EEG: *p* = 0.850; FDG-PET: *p* = 0.615), or overall immunomodulatory treatment response were detected (*p* = 0.245). The number of CSF overall alterations (*p* = 0.003) and inflammatory pathologies (*p* = 0.001) differed significantly between the subgroups, with the most frequent changes in seronegative patients with potential psychiatric autoimmune syndromes and patients with Abs against cell surface antigens.

## Discussion

In this nationwide retrospective cohort analysis, 91 patients considered by treating physicians to have AE with predominant psychiatric manifestations are reported.

In line with previous reports, on a syndrome level, the most common forms of psychiatric presentation of pAE were paranoid-hallucinatory (in 53%; [3, 4, 7]) followed by affective syndromes (in 27%). Therefore, the current study underlines that patients with possible/probable pAE can also manifest with depressive or manic disorders. Importantly, a positive history of mental disorders was reported in 45% of the patients. This finding illustrates that AE might be camouflaged by a previous history of classical idiopathic psychiatric presentation. The patients had most frequently been diagnosed with depressive disorders according to their past psychiatric histories; therefore, preexisting mental disorders definitely do not argue against pAE.

Of all patients, 21 (23%) had well-characterized neuronal Abs and fulfilled our criteria for probable pAE. Disorientation, impaired memory, and trending optical hallucinations were more frequently observed in patients with probable pAE than in patients with possible pAE. Disorientation and impaired memory are established red flag signs [11–13]. Visual hallucinations could constitute another potential red flag indicating an autoimmune cause in patients with mental disorders [14].

The most frequent Ab among the probable pAE group was against anti-NMDA-R (21% of the entire cohort), which was in two thirds of anti-NMDA-R Ab positive patients in the context of definite anti-NMDA-R encephalitis (with Ab detection in the CSF). This is a considerably higher percentage than has previously been reported [2], which is most likely due to the inclusion criteria of the current study and might point towards a selection bias. In the presented cohort, patient selection was based on suspected AE with isolated psychiatric syndromes, unlike prior reports that analyzed nonselected neurological patients and found frequencies at 4% of psychiatric anti-NMDA-R encephalitis cases [2]. Other Ab-defined probable pAEs were even rarer in our cohort. One patient was identified to have anti-LGI1 Abs. Anti-LGI1 encephalitis mostly affects older patients of both sexes and manifests with subacute to chronic memory dysfunction and personality changes with often unrecognized epileptic syndromes, such as faciobrachial dystonic seizures. Importantly, 10% of the patients with anti-LGI1 encephalitis appear to have normal MRI and CSF studies, which makes this subtype especially challenging and necessary to incorporate into differential diagnosis of suspected neurodegenerative dementias [15]. One patient had anti-MOG Abs, which are known to sometimes cause cortical encephalitis and can manifest like AE, especially in children [16, 17]. This is relevant because anti-MOG Abs are most likely not routinely examined in the diagnostic workup of patients with purely psychiatric symptoms. Importantly, commercially available anti-MOG Ab tests are less sensitive than so-called live-cell assays, while the latter are less specific, so care needs to be taken to prevent false positives and false-negatives in low prevalence settings, such as in psychiatric patients [18].

In the possible pAE group, two patients with anti-Yo Abs and prominent psychiatric symptoms were described. This is unusual, since anti-Yo Abs usually cause relentlessly progressive cerebellar degeneration, often with underlying ovarian or breast cancer, which is unlikely to be confused with psychiatric disorders [19]. However, psychiatric “extra-cerebellar” manifestations have been described [20]. Similarly, anti-Zic4 Abs are rare Abs that target an intracellular antigen and associate with underlying tumors and brain stem or cerebellar syndromes [21]. A false positive signal in the line blot cannot be excluded for anti-Yo and anti-Zic4 Ab findings. In cases of a positive line blot result, result verification using indirect immunofluorescence on brain tissue should be required. This corroboration was only documented in one of three patients. However, due to the anonymized study design, verifying whether these investigations had been performed in the other two patients was impossible. Because of these methodological ambiguities and the atypical clinical manifestations, the three patients were assigned to the possible pAE group. Patients with anti-VGKC Abs on radioimmunoassays without concomitant detection of CASPR2 or LGI1 Abs were classified as possible rather than probable pAE cases, because this finding lacks a high specificity for autoimmunity [22, 23]. Therefore, in the absence of further supportive criteria, such as CSF pleocytosis or MRI changes, the relevance of isolated anti-VGKC Abs should be regarded with caution. In contrast, the detection of anti-glycine Abs, anti-GAD65 Abs, and tissue-based detection of hippocampal neuropil-targeting Abs most likely carry a higher chance of underlying autoimmunity; however, these patients were also considered as possible but not probable pAE cases because their specificity is still smaller than the “well-characterized” Abs described above [24–27]. Overall, 70 patients (77%) were identified who had nonspecific findings suggestive of underlying AE in Ab testing, EEG, CSF, or neuroimaging that were classified as possible pAE.

CSF and MRI abnormalities (in 77% and 57%, respectively) were the most frequent alterations in patients with possible and probable pAE. Indeed, it was previously shown that inflammatory CSF changes and MRI abnormalities are not uncommon in patients with psychiatric syndromes and should trigger suspicion for underlying autoimmunity [4, 28, 29]. White matter lesions were identified most frequently (49%) via MRI; such lesions need not necessarily be signs of an inflammatory process but could also be signs of cerebral microangiopathy. Nevertheless, no correlation of white matter lesions with age existed, which does not support the idea of age-related microangiopathic changes. Brain FDG-PET investigations were acquired only in 30% of the patients (mostly in patients with nonspecific EEG, MRI, or CSF changes) and were found to be abnormal in 56% of cases. Previous studies on AE have shown that FDG-PET could be more sensitive than MRI in detecting AE [30–32]. FDG-PET can play a supportive role in detecting AEs, especially in situations in which available clinical and diagnostic findings are inconclusive (e.g., [5, 6]). In summary, the current data highlight the importance of comprehensive diagnostic workup in patients with severe mental disorders, especially if patients present with “red flag” symptoms [7, 11–13]. However, they also show that patients with psychiatric syndromes suspected to be of immune-mediated origin are often seronegative for known neuronal Abs.

While treatment decisions in seropositive syndromes are supported by expert recommendations [1]—albeit from retrospectively generated evidence—the choice of immunotherapy in possible pAE is considerably more challenging. In the current study, a majority of the patients with suspected AE was treated with immunotherapies. Most frequently, steroids, plasmapheresis, and IVIGs were used for treatment. However, B-cell depletion using rituximab was initiated only in 14% of patients with probable pAE and in 1% with possible pAE. Patients without well-defined Abs were more likely to be treated with oral steroids and steroid-sparing agents. Altogether, 83% of the patients were considered to have benefitted from this treatment.

Notably, only treating physician-based assessments of treatment response was acquired, but neither a systematic follow-up nor standardized or quantitative outcome data were available. Furthermore, the placebo effect of immunotherapy has to be considered. Therefore, the response to therapy is likely to be biased. Nevertheless, it is worth noting that no significant differences between patients with probable and possible pAE were observed in terms of their responses to treatment. This supports the notion that immunotherapy should not be limited to patients with currently known neuronal Abs but could also be considered for seronegative patients with sufficient evidence of AE. In line with this observation, some patients also harbored Abs against uncharacterized neuronal antigens, and such cases illustrate that novel Abs could possibly play a relevant role in a subgroup of patients with psychiatric syndromes (cf. [5]).

This cumulative collection of anonymous patient cases with an open, unsystematic, cross-sectional, and uncontrolled approach has some major limitations. A selection bias exists, since successfully treated patients were more likely to be included in the case collection and clinical improvement as a result of immunotherapy was even one of several potential inclusion criteria. Therefore, and because of unrecognized cases, the prevalence of AE in psychiatric inpatients cannot be estimated from this data and the significance of the reported therapy response should not be generalized. The test methods were not standardized (analysis in diverse laboratories with differences in standard operating procedures, different MRI sequences, FDG-PET only available in a subgroup of patients, etc.) and sometimes not clearly comparable due to this heterogeneity [33]; however, this represents the real-world situation. In addition, diagnostic workup to exclude differential diagnoses was not harmonized between centers. Rather it illustrates how different the diagnostic procedures are at different hospitals. Therefore, this project highlights that there is an absolute need for a more standardized procedure in clinical routine. The inclusion criteria were defined very broadly to avoid missing borderline psychiatric cases (e.g., neuronal Ab detection in serum was sufficient for inclusion and detection in CSF was not explicitly required) and were not identical to the criteria for neurological patients by Graus et al. [1]. However, that the proposed classification into possible pAE and probable pAE allows a gradation of the certainty of an AE is supported by findings such as a 1.6-fold less frequent occurrence of an increased WBC count in the possible pAE compared with the probable pAE group. In addition, psychiatric preexisting conditions were twice as common in the patients with possible pAE, which could also argue for some false positives with AE mimics, especially in the cohort with possible pAE. In clinical practice, care must be taken to nonspecific findings such as increased CSF protein levels or microangiopathic white matter lesions in MRI frequently found in the general population and could be interpreted as suggestive of brain autoimmunity in patients with psychiatric symptoms, thereby leading to false conclusions. Finally, the lack of follow-up and long-term outcome data has most likely biased the current data on the response to therapy. Nevertheless, our data provide an important cross-sectional insight into the characteristics of psychiatric patients suspected of having pAE.

## Conclusions

The main conclusion regards the need for extensive diagnostic workup in patients with psychiatric syndromes, especially in those with red flag symptoms, including cerebral MRI, EEG, CSF analyses, and sometimes brain FDG-PET. Such a process has been proposed by the German consensus and evidence-based schizophrenia guidelines [34, 35] but is still neither universally adhered to nor an international standard. Such diagnostic schemes are not only important for identifying patients in whom to consider comprehensive Ab studies for neuronal (and possible antiglial MOG) Abs but also a considerable proportion of patients diagnosed (as possible pAE) based on nonspecific MRI, CSF, and/or EEG findings. These patients could benefit from immunotherapy, although exact treatment regimens and types are largely unclear. However, no findings in these latter diagnostic procedures are by themselves specific to underlying autoimmunity, and even short-term immunosuppression can have considerable side effects. At the same time, lack of clinical improvement under short-term immunotherapy does not exclude an autoimmune etiology. Therefore, prospective trials evaluating therapy responses and patient outcomes within predefined patient cohorts with a high likelihood of underlying autoimmunity should be performed. Until then, utmost caution is needed to prevent not only undertreatment but also overtreatment of currently seronegative AE patients with purely psychiatric manifestations.

## Supplementary information

Supplemental Table 1
