# Supplementary material for: Clinical manifestations and immunomodulatory treatment experiences in psychiatric patients with suspected autoimmune encephalitis: a case series of 91 patients from Germany
Source: Mol Psychiatry. 2022 Jan 19;27(3):1479–89. doi: 10.1038/s41380-021-01396-4 (PMC9095476; doi:10.1038/s41380-021-01396-4)
Supplement: Supplementary file 1 — Supplemental Table 1 [file 41380_2021_1396_MOESM1_ESM.pdf]

	**Abs against neuronal cell surface antigens (N=35)***	**Abs against neuronal intracellular antigens (N=9)**	**Probable Hashimoto encephalopathy (N=14)**	**Seronegative potential psychiatric autoimmune syndromes based on MRI, CSF/EEG findings or therapy (N=33)**	**Statistics**
**Age**	37.79±16.85	36.33±19.02	45.07±14.72	42.56±14.79	F=1.074, p=0.365
**Sex** (female:male)	57% (20):43% (15)	44% (4):56% (5)	93% (13):7% (1)	45% (15):55% (18)	Chi²=9.725, **p=0.021**
**Syndromes**					Chi²=20.187, p=0.322
Paranoid-hallucinatory	57% (20/35)	44% (4/9)	64% (9/14)	45% (15/33)	
Depressive	11% (4/35)	33% (3/9)	14% (2/14)	36% (12/33)	
Manic	6% (2/35)	11% (1/9)	7% (1/14)	0% (0/33)	
Dementing/cognitive dysfunction	14% (5/35)	0% (0/9)	7% (1/14)	9% (3/33)	
Confusional/delirious	3% (1/35)	0% (0/9)	7% (1/14)	9% (3/33)	
Personality change	6% (2/35)	0% (0/9)	0% (0/14)	0% (0/33)	
Other	3% (1/35)	11% (1/9)	0% (0/14)	0% (0/33)	
**Diagnostic alterations**					
MRI	55% (18/33)	33% (3/9)	69% (9/13)	61% (20/33)	Chi²=3.102, p=0.376
EEG	43% (12/28)	50% (4/8)	40% (4/10)	34% (10/29)	Chi²=0.796, p=0.850
FDG-PET	50% (6/12)	50% (2/4)	100% (2/2)	56% (5/9)	Chi²=1.800, p=0.615
CSF overall	72% (23/32)	56% (5/9)	54% (7/13)	97% (31/32)	Chi²=13.774, **p=0.003**
CSF inflammatory changes**	59**%** (19/32)	22% (2/9)	15% (2/13)	72% (23/32)	Chi^2=15.017, **p=0.001**
**Immunomodulatory treatment**					
Overall improvement	77% (20/26)	67% (2/3)	100% (14/14)	80% (12/15)	Chi²=4.162, p=0.245

Supplemental Table 1: Subgroup descriptions. *Including patients with not well-characterized neuronal autoantibodies against neuronal cell surface antigens such as anti-glycine autoantibodies or novel neuronal autoantibodies on tissue tests. **Inflammatory CSF changes were defined as increased CSF white blood cell count and/or CSF specific oligoclonal bands. Abbreviation: Abs, autoantibodies; Ab, autoantibody; CSF, cerebrospinal fluid; EEG, electroencephalography; FDG-PET, [^{18}F]fluorodeoxyglucose positron emission tomography; MRI, magnetic resonance imaging.
